# The dogma of aspirin: a critical review of evidence on the best monotherapy after dual antiplatelet therapy

**DOI:** 10.1186/s12959-015-0059-8

**Published:** 2015-09-07

**Authors:** Hernan Polo Friz, Mauro Molteni, Claudio Cimminiello

**Affiliations:** Department of Medicine, Vimercate Hospital Azienda Ospedaliera di Desio e Vimercate, Vimercate, Italy

**Keywords:** Platelet aggregation inhibitors, Evidence-based practice, Aspirin, Clopidogrel

## Abstract

Dual antiplatelet therapy based on the combination of an adenosine diphosphate (ADP)-receptor antagonist plus aspirin has demonstrated to be more effective in reducing the rate of major ischemic vascular events compared to aspirin monotherapy in some clinical settings. The current controversy on the duration of dual antiplatelet therapy should not conceal another major issue: the choice of the more appropriate antiplatelet monotherapy after the dual treatment phase. The aim of this article is to critically analyze the available evidence in this topic.

Data from studies like CAPRIE, MATCH, PROFESS, CHANCE, DAPT and others, raise questions as why antiplatelet monotherapy after the dual phase should only be based on aspirin, in spite of a lack of evidence surprisingly not highlighted by key opinion leaders and experts.

We conclude that, whether ADP-receptor antagonist rather than aspirin may be proposed as monotherapy seems not only have no answer but also not place in the current specialists’ analysis, as if a dogmatic approach were prevalent. Perhaps the time for an open debate on these topics is ripe.

## Introduction

At the end of the 90s dual antiplatelet therapy based on the combination of an ADP-receptor antagonist plus aspirin has been proposed to reach a more efficient inhibition of platelet function by simultaneously blocking different platelet-activation pathways [1]. This approach demonstrated to be more effective in reducing the rate of major ischemic vascular events compared to aspirin monotherapy, but only in some clinical settings. For instance, in coronary artery disease (CAD), dual antiplatelet therapy with aspirin plus clopidogrel has long been considered the standard of care for treating acute coronary syndrome (ACS) and after percutaneous coronary intervention (PCI) since its benefits over aspirin monotherapy has been well established [2–7]. In patients with acute ischemic stroke and in subjects with symptomatic peripheral arterial disease (PAD), even recent guidelines did not recommend (or did it only for selected patients) the combination of an ADP-receptor antagonist plus aspirin based on evidence apparently not in favor of this choice [8, 9]. However, CHANCE trial showed that therapy with clopidogrel plus aspirin initiated early after ischemic stroke or transient ischemic attach (TIA) may further reduce recurrent stroke and major vascular events compared to aspirin monotherapy [10, 11]. Indeed, dual antiplatelet therapy associates with an increased risk of bleeding. Therefore, the short-term benefits and harms of dual antiplatelet therapy observed in patients with ACS, those undergoing PCI, and those with ischemic stroke or TIA, are not directly applicable to long-term therapy and treatment duration is still a controversial issue.

Thus, research is currently trying to determine which is the optimal duration of dual antiplatelet therapy in the setting where it showed a favorable clinical impact. However these efforts of the scientific community should not conceal another major issue related to antiplatelet therapy. That is, the choice of the more appropriate antiplatelet monotherapy after the dual treatment phase. The Global Leaders study conducted with ticagrelor, clopidogrel and aspirin after a stent procedure will address this question (ClinicalTrials.gov Identifier: NCT01813435).

In the meantime the analysis of available evidence may help to better understand it.

## Review

### Dual and mono antiplatelet therapy after TIA and ischemic stroke

In 1996, the CAPRIE trial [12] showed that clopidogrel was marginally but significantly better than aspirin in patients with recent ischemic stroke, recent myocardial infarction (MI), or symptomatic PAD, reducing the relative risk for the primary endpoint (ischaemic stroke, MI, or vascular death) by 8.7 % versus aspirin. For the subgroup of patients with ischemic stroke the relative risk reduction was 7.3 % (not significant) even though the study was not designed to specifically address this subgroup of patients. Afterwards, other randomized controlled trials in patients with coronary manifestations of atherothrombosis were showing the sustained benefit of clopidogrel on top of standard treatment including aspirin, with an acceptable increase in the risk of major bleeding complications [13, 14]. Therefore, the MATCH (Management of ATherothrombosis with Clopidogrel in High-risk patients) study was performed, with the aim to find out whether aspirin added to clopidogrel as compared to clopidogrel alone would further reduce the risk of recurrent ischaemic vascular events in high-risk patients after TIA or ischaemic stroke [15]. Conclusion was that although mortality was the same in both groups and no significant increase in fatal bleeding was recorded, addition of aspirin to clopidogrel resulted in a significantly higher bleeding rate that offset the beneficial effect. Adding aspirin to clopidogrel provided no further benefit, while increasing the harm. However, in the MATCH trial less than 20 % of patients were enrolled within 7 days from stroke onset, indicating that the trial largely missed the period when the risk is high and the treatment effect would be greatest. Moreover, more than 50 % of the patients had an etiologic mechanism of small-vessel occlusion, which associates to major bleeding, decreasing the benefit of clopidogrel and aspirin dual therapy. Furthermore, a cause of major or life-threatening bleeding that was increased by adding aspirin to clopidogrel was gastrointestinal bleeding, probably indicating the known deleterious effect of aspirin on the gastrointestinal mucosa [16]. Interestingly enough, MATCH study results were most considered evidence against dual therapy (or failure to demonstrate the benefits) instead of hypotheses generating data on a small but definitely superior benefit of clopidogrel over aspirin as monotherapy for stroke. On the contrary, some authors have questioned on what pushed clinical neurologists from 507 centers in 28 countries participating in the study MATCH to accept the experimental trial design which provided for clopidogrel the role of reference therapy in place of asp2irin. It’ easy to answer this question by recalling that in those years was emerging that the benefit of aspirin monotherapy for secondary prevention in patients with ischemic stroke or TIA was much less than expected for antiplatelet therapy as a whole [17]. These data, together with those of Hankey et al. in his Cochrane review published roughly at the same time on the modestly but significantly better efficacy of ADP-receptor antagonists as compared with aspirin in stroke patients [18] constituted a valid reason for all those neurologist involved in MATCH to try to offer their patients with stroke something more effective than aspirin monotherapy.

**Fig. 1:**
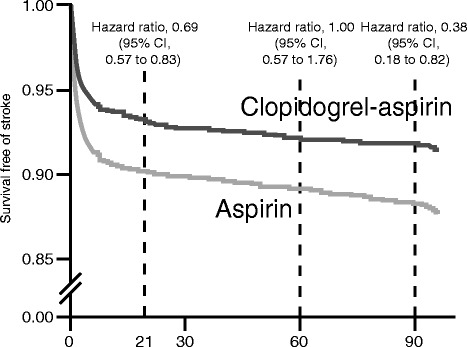
Risk of stroke during different phases (first 21 days, from 22nd to 60th day, from 61st to 90th day) of the CHANCE study [10, 11] follow-up period. Overall benefit in favour of aspirin-clopidogrel: hazard ratio, 0.68 (95 % CI, 0.57–0.81) *P* < 0.001

In 2013, Wang and colleagues reported the results of the Clopidogrel in High- Risk Patients with Acute Non disabling Cerebrovascular Events (CHANCE) trial [10, 11] which enrolled 5170 patients with acute minor ischemic stroke or TIA at high risk for recurrence and, unlike MATCH study, within 24 h of symptom onset. The addition of clopidogrel to aspirin in comparison to aspirin alone reduced the relative risk of recurrent stroke at 90 days by 32 % (8.2 % vs. 11.7 %; hazard ratio, 0.68; 95 % CI, 0.57 to 0.81) with no difference between the group that received both groups in the incidence of moderate or severe hemorrhage (0.3 % in each group; *P* = 0.73) or hemorrhagic stroke (0.3 % in each group; *P* = 0.98). The study demonstrated a substantial treatment effect with a number-needed-to-treat of 29 for preventing one recurrent stroke. That is, treating 29 patients for 90 days with clopidogrel plus aspirin for the first 21 days, followed by clopidogrel alone from day 22 to day 90, prevented one stroke, as compared with aspirin alone. Is noticeable that most of the absolute benefit of clopidogrel plus aspirin is obtained within the first few days after the ischemic event. The strategy adopted in the CHANCE study to employ dual antiplatelet therapy for less than a month and then switch to a single agent is proof of how the lesson of MATCH has been carefully read thus avoiding to pay a too high tribute in terms of intracranial hemorrhage since in MATCH intracranial hemorrhages were becoming significantly more frequent in the group of combined therapy after the first quarter [19, 20]. The benefit shown by clopidogrel monotherapy compared with aspirin in the days between 22 and 90 once again underlines, in an unbiased reading of CHANCE, the issue already emerged in the MATCH of clopidogrel as better candidate for antiplatelet monotherapy after the dual phase, Fig. 1, and the PROFESS findings still in patients with ischemic stroke are consistent with this evidence [21].

### Dual and mono antiplatelet therapy after PCI

**Fig 2:**
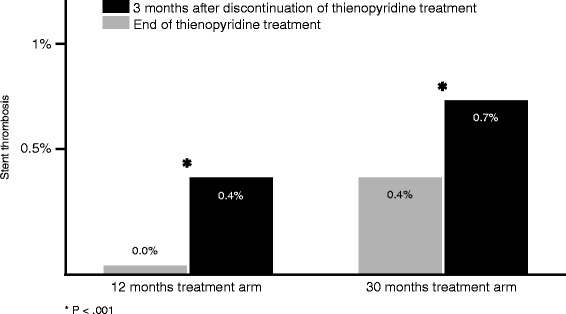
DAPT study [24]: risk of stent thrombosis in patients treated for 12 and 30 months during the 3 months after discontinuation of thienopyridine treatment

With regard to antiplatelet therapy following PCI, the use of dual therapy is critically important for the prevention of coronary stent thrombosis, and this therapy is currently recommended for 6 to 12 months after implantation of a drug-eluting stent (DES) [22, 23]. However, the optimal duration remains unknown. To test the hypothesis that a 30-month duration of therapy with aspirin plus an ADP-receptor antagonist would be superior to a 12-month duration in patients undergoing PCI, DAPT trial was carried out and results recently published by Mauri et al. [24]. All patients received aspirin with 5020 patients randomized to prolonged DAPT and 4941 to placebo. Approximately two thirds of the patients received clopidogrel, whereas the rest received prasugrel. The primary endpoint of major adverse cardiac and cerebrovascular events was significantly lower in the continued DAPT arm compared with placebo (4.3 % vs. 5.9 %, hazard ratio 0.71, 95 % confidence interval 0.59–0.85, *p* < 0.001). There were reductions in all MI (2.1 % vs. 4.1 %, *p* < 0.001) and stent thrombosis (0.4 % vs. 1.4 %, *p* < 0.001), but all-cause mortality was higher (2.0 % vs. 1.5 %, *p* = 0.05), driven mostly by an increase in non-cardiovascular deaths (1 % vs. 0.5 %, *p* = 0.002), including cancer-related death (0.62 % vs. 0.28 %, *p* = 0.02) and bleeding-related death (0.22 % vs. 0.06 %, *p* = 0.06). GUSTO moderate and severe bleeding was also higher with DAPT (2.5 % vs. 1.6 %, *p* = 0.001). The results of the DAPT trial indicate that prolonged duration of DAPT up to 30 months following index PCI with a DES results in lower stent thrombosis and recurrent MIs compared with a 12-month duration of DAPT, although bleeding and all-cause mortality were higher with prolonged therapy. The excess in mortality is concerning, but appears to be a combination of cancer-related and bleeding-related mortality. However, an important finding of the study risks of going unnoticed. In the mentioned trial, the interruption of ADP-receptor antagonists therapy both in the group of early discontinuation and in those receiving extended dual therapy was followed in the next quarter by an important increase of stent thrombosis and MI, suggesting that an ADP-receptor antagonist instead of aspirin might be the best choice as monotherapy after dual phase antiplatelet treatment, Fig. 2. Yet is easy to refer to the evidence which made possible [24, 25] the large-scale use of coronary stents in clinical practice thanks to the addiction of ADP-receptor antagonists to aspirin which alone had failed in preventing stent occlusion. The question that arises from DAPT and a number of previous studies [26] is why antiplatelet monotherapy after the dual phase should only be based on aspirin? What is the evidence supporting that patients after a period of dual antiplatelet therapy for having received a coronary stent must return to antiplatelet monotherapy with aspirin? Instead, in these patients it may make sense to think about the use of an ADP-receptor antagonist rather than aspirin as monotherapy.

### Dual and mono antiplatelet therapy in patients with PAD

In contrast to coronary artery disease and ischemic stroke, there have not been trials with sufficient power to estimate precisely the preventive effects of antiplatelet drugs in patients with PAD. A meta-analysis compared the efficacy of antiplatelet therapy in approximately 135,000 high-risk patients with vascular diseases, including 9214 patients with lower extremity PAD. Among those patients with PAD treated with antiplatelet therapy there was a 23 % relative risk reduction (*p* < 0.004) of adverse cardiovascular events, like MI, stroke, or vascular death. However, only one third of the patients were treated with aspirin, and using different dosages [27].

A more recent recent metanalysis performed by Berger et al. [28] on 5269 subjects with PAD investigated the efficacy of aspirin alone or the combination of aspirin and dipyridamole. In the subset of 3019 participants taking aspirin alone vs control, aspirin was associated with a non-significant reduction in cardiovascular events (RR, 0.75; 95 % CI, 0.48–1.18). On the other hand, in the subgroup of patients with PAD in the CAPRIE study, a reduction of cardiovascular events was observed in clopidogrel-treated patients compared to those who received aspirin. The reduction, deriving from a prespecified analysis and resulting from a stratified randomization for the clinical condition of PAD, was 22.8 % and was consistent with the overall direction of the study results.

In aggregate these evidences are unable to state what is the best option for the cardiovascular prevention in PAD patients but perhaps a preference could come for clopidogrel. However, in spite of scanty and inconclusive data on the efficacy of aspirin in cardiovascular prevention in PAD patients aspirin remains the most used drug for management of these patients. Yet, the American College of Chest Physicians (ACCP) guidelines recommend aspirin (75–100 mg/day) or clopidogrel (75 mg/day) for asymptomatic PAD patients (including patients with peripheral arterial bypass surgery or percutaneous transluminal angioplasty) as equivalent drugs and without limitation of clopidogrel use (Grade 1A) [8] and the 2013 guidelines on the management of patients with PAD by the American College of Cardiology Foundation/American Heart Association recommend Aspirin, typically in daily doses of 75 to 325 mg, as safe and effective antiplatelet therapy to reduce the risk of MI, stroke, or vascular death in individuals with symptomatic atherosclerotic lower extremity PAD and clopidogrel (75 mg per day) as a safe and effective alternative, with the same level of evidence than aspirin [9].

## Conclusions

Evidence on the absolute benefits of dual antiplatelet therapy early after ACS, and PCI and also after ischemic stroke is clear. However focusing on optimal duration of dual therapy should not distract the scientific community from the very important issue of the choice of the more appropriate antiplatelet monotherapy after the dual treatment phase, or as monotherapy in PAD patients. If a blinded experiment would prove that the combination of drugs A + B is not more effective than just drug A but causes more adverse reactions, everyone would agree with the futility of drug B. What emerged from a study considered almost heretical as the MATCH is that but the use of aspirin–in that study the drug B–has never been discussed as it should have been.

Aspirin, as the oldest antiplatelet drug, seems to be accepted as the only option for the management of these patients, in spite of a lack of evidence surprisingly not highlighted by key opinion leaders and experts.

Questions as why antiplatelet monotherapy after the dual phase should only be based on aspirin or whether use of ADP-receptor antagonists rather than aspirin may be proposed as monotherapy especially now that clopidogrel is available as a generic drug, seems not only have no answer but also not place in the current specialists’ analysis. As if a dogmatic approach were prevalent. Perhaps the time for an open debate on these topics is ripe.

## Abbreviations

ADP: Adenosine diphosphate
CAD: Coronary artery disease
ACS: Acute coronary syndrome
PCI: Percutaneous coronary intervention
PAD: Peripheral arterial disease
TIA: Transient ischemic attach
MI: Myocardial infarction
DES: Drug-eluting stent
